# Leishmaniasis and phlebotomine sand flies in Oman Sultanate

**DOI:** 10.1051/parasite/2020064

**Published:** 2020-11-27

**Authors:** Jean-Antoine Rioux, Marina Gramiccia, Nicole Léger, Philippe Desjeux, Jérôme Depaquit

**Affiliations:** 1 Faculté de Médecine, Université Montpellier 1 1 rue de l’Éencole de Médecine 34000 Montpellier France; 2 Department of Infectious Diseases, Unit of Vector-borne Diseases, Istituto Superiore di Sanità Viale Regina Elena 299 00161 Rome Italy; 3 Université de Reims Champagne-Ardenne, EA7510, Université de Reims Champagne-Ardenne, Faculté de Pharmacie 51 rue Cognacq-Jay 51096 Reims cedex France; 4 PATH OWH (formerly One World Health) A-9, Qutub Institutional area, USO Road New Delhi 110067 India; 5 ANSES, USC Transmission Vectorielle et Épidémiosurveillance de Maladies Parasitaires (VECPAR) 51100 Reims France; 6 Laboratoire de Parasitologie, Pôle de Biologie, Centre Hospitalier Universitaire de Reims 51100 Reims France

**Keywords:** Eco-epidemiology, *Leishmania* isolation and typing, Patients, Phlebotomine sandflies, Oman

## Abstract

There are few data on leishmaniases and sandflies in Oman Sultanate. We carried out an eco-epidemiological study in 1998 in the two main mountains of the country, the Sharqiyah and the Dhofar. This study allowed us to isolate and identify three *Leishmania* strains from patients exhibiting cutaneous leishmaniasis. The typing carried out by isoenzymatic study and by molecular biology were congruent: two strains of *Leishmania donovani* zymodeme (Z) MON-31 isolated in the Sharqiyah and one *L. tropica* ZROM102 (ZMON-39 variant for 4 isoenzymes) from the Dhofar. No strain was isolated from canids. The study of sandflies identified 14 species distributed in the genera *Phlebotomus*, *Sergentomyia* and *Grassomyia*: *Ph. papatasi*, *Ph*. *bergeroti*, *Ph*. *duboscqi*, *Ph*. *alexandri*, *Ph*. *saevus*, *Ph*. *sergenti*, *Se. fallax*, *Se*. *baghdadis*, *Se*. *cincta*, *Se*. *christophersi*, *Se*. *clydei*, *Se*. *tiberiadis*, *Se*. *africana*, and *Gr. dreyfussi*. In Sharqiyah, the only candidate for the transmission of *L. donovani* was *Ph. alexandri*, but the low densities observed of this species do not argue in favor of any role. In Dhofar, *Ph. sergenti* is the most important proven vector of *L. tropica*, but *Ph. saevus*, a locally much more abundant species, constitutes a good candidate for transmission.

## Abstract (fr)

Il existe peu de données sur les leishmanioses et les phlébotomes en Oman. Nous y avons mené en 1998 une étude éco-épidémiologique dans les deux principaux massifs montagneux du pays, la Sharqiyah et le Dhofar. Cette étude nous a permis d’isoler et d’identifier trois souches de *Leishmania* à partir de patients présentant des leishmanioses cutanées. Les typages menés par étude isoenzymatique et par biologie moléculaire ont été congruents : deux souches de *Leishmania donovani* ZMON-31 isolées dans la Sharqiyah et une de *L. tropica* ZROM102 (ZMON-39 variant pour 4 isoenzymes) originaire du Dhofar. Aucune souche n’a été isolée à partir de Canidés. L’étude des Phlébotomes a permis d’identifier 14 espèces réparties dans les genres *Phlebotomus*, *Sergentomyia* et *Grassomyia* : *Ph. papatasi*, *Ph*. *bergeroti*, *Ph*. *duboscqi*, *Ph*. *alexandri*, *Ph*. *saevus*, *Ph*. *sergenti*, *Se. fallax*, *Se*. *baghdadis*, *Se*. *cincta*, *Se*. *christophersi*, *Se*. *clydei*, *Se*. *tiberiadis*, *Se*. *africana* et *Gr. dreyfussi*. Dans la Sharqiyah, la seule espèce candidate à la transmission de *L. donovani* est *Ph. alexandri* mais les faibles densités observées de cette espèce ne plaident pas en faveur d’un quelconque rôle. Dans le Dhofar, *Ph. sergenti* est le principal vecteur prouvé de *L. tropica* mais *Ph. saevus*, espèce localement bien plus abondante, constitue une bonne espèce candidate à la transmission.

## Introduction

Leishmaniasis remains poorly documented in Oman Sultanate as well as the fauna of their vectors, Phlebotomine sandflies [5]. A few case reports are available in the literature of both visceral (VL) [6, 12, 21–23, 30, 63, 64] and cutaneous (CL) [64, 65, 71] leishmaniases. However, to our knowledge, no parasite has been cultured and typed according to gold standard methods (isoenzymes or PCR-RFLP or sequencing of targeted markers) except one strain of *L. tropica* isolated from a Pakistani patient continuously resident in Oman for the 18 months before parasite isolation [65]. No record of affected animals like dogs has been documented. The agents of VL belong to the *L. donovani* complex without identification at the specific level (*L. donovani s. st.* or *L. infantum*) [63]. A few studies have been carried out to identify the sandflies of the Sultanate [36].

Visceral leishmaniasis is confined principally to children in the Sharqiyah [50] and Dhofar [25] governorates. Between 1992 and 1995, the annual incidence rate of VL in Oman varied from 14 to 40 cases, but many children treated empirically for kala-azar are not reported [64]. The annual incidence rate is decreasing to 15–20 CL and 2–4 VL cases yearly [8].

The goal of the present study was to isolate and culture *Leishmania* strains in order to identify them from humans and wild or domestic canids, and to carry out an inventory of the Phlebotomine sandflies of the country in order to determine candidate(s) for *Leishmania* transmission according to the eco-epidemiological concept. This concept started in the 1950s and was applied in Mediterranean foci (France, Italy, Spain, Tunisia, Algeria, Morocco, Syria) and the Arabian peninsula (Yemen).

## Material and methods

### Ethical approval

The study (inclusion of patients, animals and captures of Phlebotomine sandflies) was carried out in 1998 in agreement with the World Health Organization, the Omani Ministry of Health, and the Omani Ministry of Agriculture. All laws and regulations were strictly followed. At that time, no ethics committee existed in Oman [7]. In all cases, patient records and information were anonymised and de-identified prior to analysis.

### Study sites

We prospected the two main biogeographical regions of Oman from September 26 to October 26, 1998: Sharqiyah and Dhofar.

In the Sharqiyah region, analysis of places where CL and VL cases occurred was performed on selected farms in the Ibra alluvial basin. Houses were occupied by one to two families living mostly with some cows and herds of sheep and goats. The cows remained in the stable, while the herds were driven into the steppes and the surrounding hills. In order to complete the vector sampling, trapping with adhesive traps was carried out on the rockslides, cracks, holes and caves of the Ouadi Mouqal cliffs.

**Figure 1:**
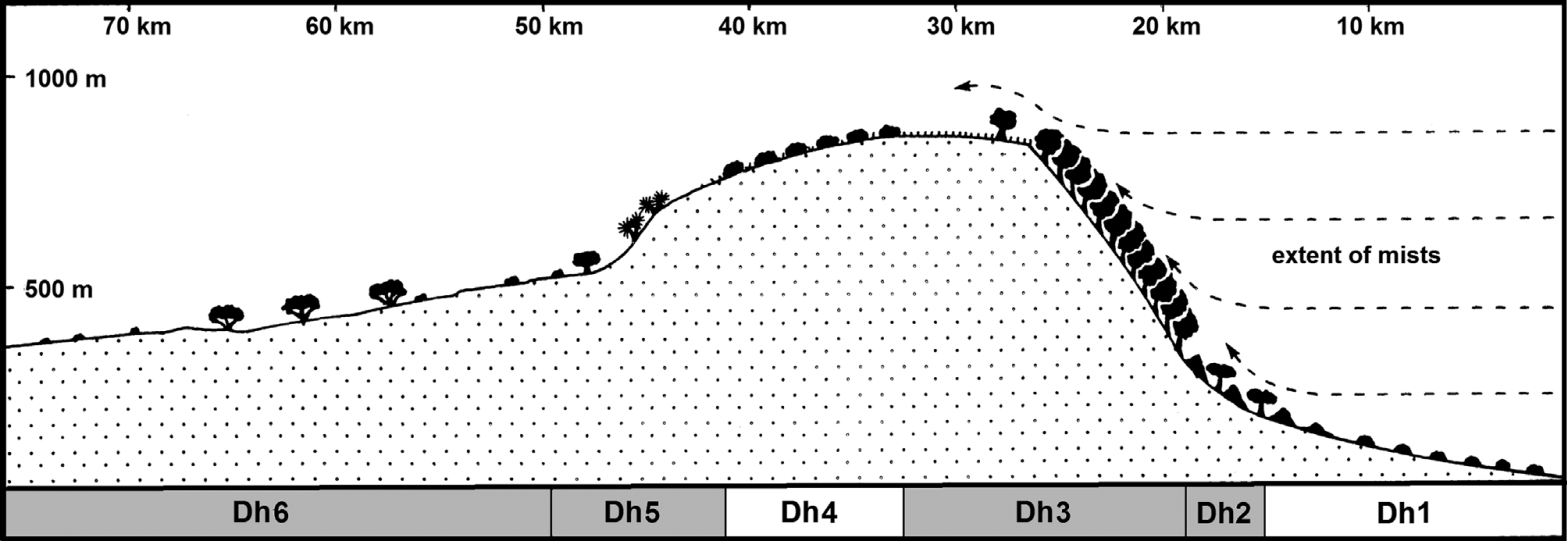
Diagram showing a cut of the Djebel Qara, in the vicinity of Salalah (Dhofar) after [46], modified. Dh1: shores with *Avicennia marina* (residual mangrove); Dh2: piedmont with *Boscia arabica*; Dh3: mountain flanks and humid escarpments with *Anogeissus dhofarica* and hilly plateaus with steppes and grasses; Dh4: arid plateau with *Euphorbia balsamifera*.; Dh5: scree and perarid reg desert with *Boswellia sacra*; Dh6: wadis and perarid cliffs with *Acacia ethbaica* and *Dracaena serrulata*. The sampled bioclimatic levels are indicated in grey.

**Figure 2:**
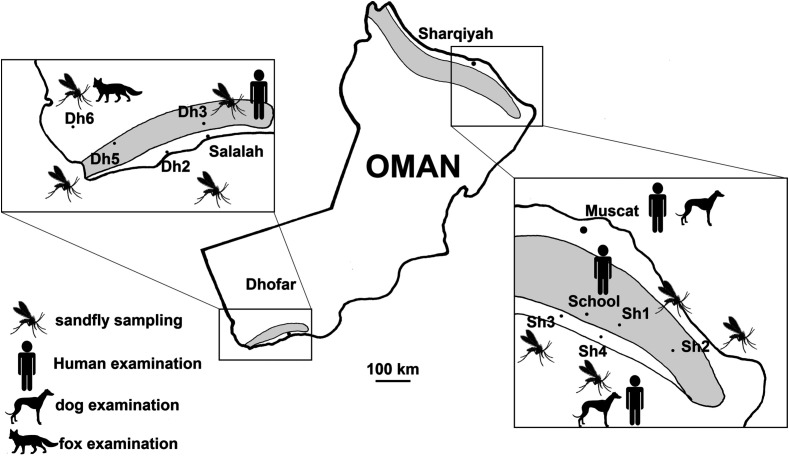
Map showing the sampling of phlebotomine sandflies, dogs, foxes and patients. Locations in the Sharqiyah are labelled Sh and those from the Dhofar are labelled Dh, in accordance with Figure 1.

**Figure 3:**
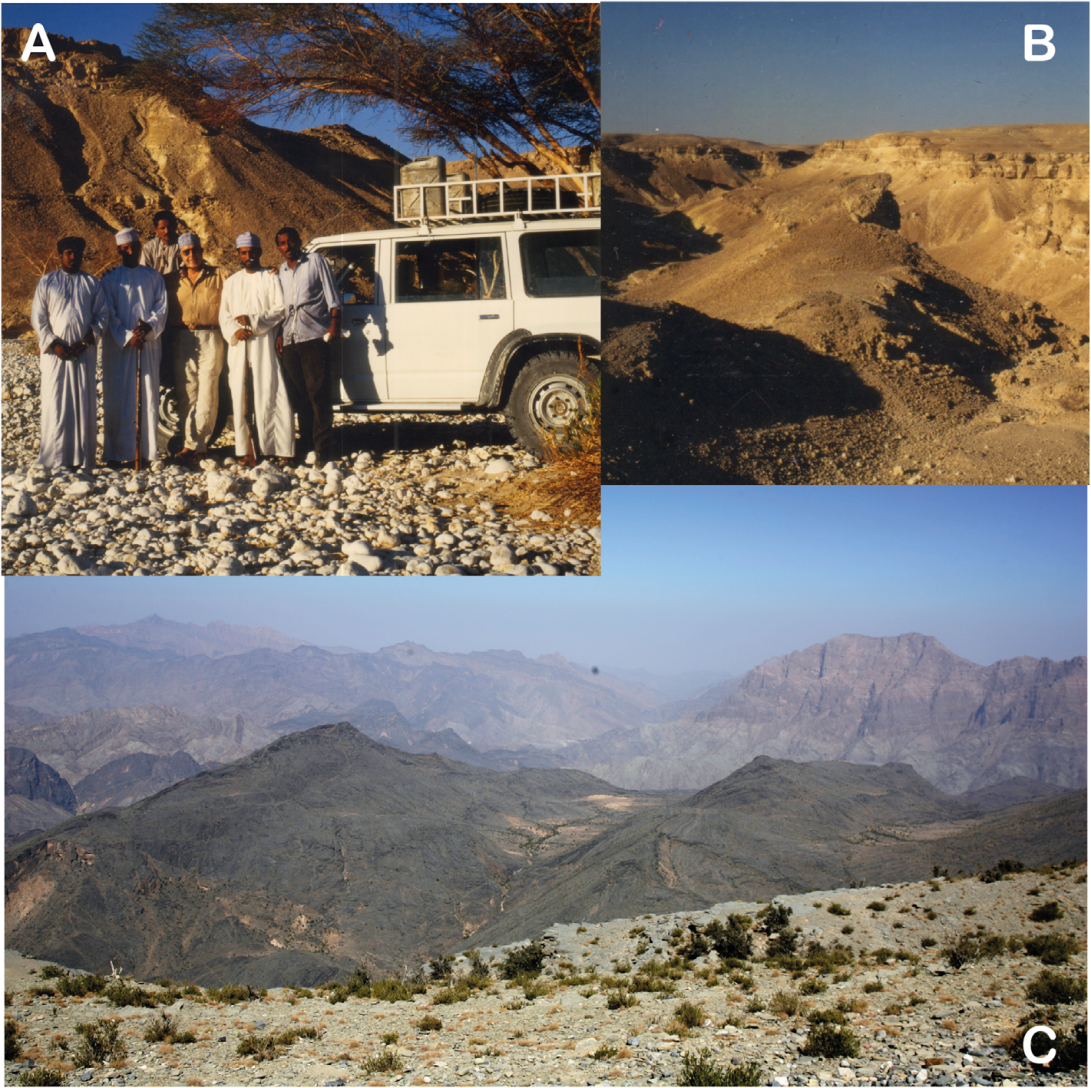
(A) Professor Rioux and the field team in the Dhofar; (B) Ouadi Herwouib in the Dhofar labelled Dh6 in accordance with Figure 1 where *Ph. bergeroti*, *Ph. alexandri*, *Se. tiberiadis* and *Se. fallax* are the most abundant species. (C) the Sharqiyah around the Ouadi.

The Dhofar region was given special attention, not only because of the presence of VL and CL, but also its phyto and zoo-geographical originality (Afro-tropical elements, endemism) [26, 46]. The use of the transect method lead us to sample the different phyto-ecological climaxes, from Salalah to Herwouib, through the Qara and Qamar djebels, the Jejouel reg, the Wad Afaoul and the Wadi Herwouib (Figs. 1, 2 and 3). Among these climaxes, two were chosen because of their endemic richness: the slopes watered with *Anogeissus* and the thalwegs and wadi with *Acacia*, *Boswellia* and *Dracaena*.

### Phlebotomine sandflies sampling

The trapping sites were selected according to both field observations (orography, geology, geomorphology, vegetation, human habitat) and information acquired prior to the field work: human cases of VL or CL, entomological and parasitological studies, and expert reports. Sampling was carried out by combining miniature CDC traps and sticky traps.

One to three CDC miniature light traps were installed in the previously selected biotopes: houses, stables, sheepfolds, caves, and various vegetation. They were placed at the end of the afternoon and picked up the next morning before sunrise. Sandflies were stored in 95% ethanol.

We express the relative frequencies of species by reporting the number of sandflies caught per “night/trap” (s/n/t).

Sticky traps were made with white paper sheets (20 × 20 cm for a double-sided active surface of 800 cm^2^) impregnated with castor oil. They are placed in the crevices of walls or rocks, at the opening of burrows, in stables, sheepfolds, and henhouses. The number of traps deposited is shown in Table 1. Sandflies are collected from the trap with a little brush and stored in 100% ethanol. After their identification, results were expressed as the number of specimens (males, females, total) per species and per square meter of trap (relative densities). The grouping of the stations allowed for calculation of the densities by climax [58, 59].

**Table 1:** Details about the places where sandflies, patients, dogs and fox have been sampled.

Symbol (Fig. 1)	Collection	Location	Latitude	Longitude	Altitude	Number of	Number of CDC traps	Number of patients examinated	Number of *Leishmania* strains isolated	Number of dogs examinated	Number of fox examinated
Sh1	Sandflies	El Kederi farm	22°47 N	58°41 E	450	33	4				
		Batem	22°49 N	58°41 E	400	20	1				
Sh2	Sandflies	Hedna	22°37 N	59°06 E	400	31	4				
		Ouali gorges	22°36 N	59°05 E	600	101					
		Ouali Mouqual	22°36 N	59°05 E	550	188					
Sh3	Sandflies, patient	Al Rawdah	22°53 N	58°13 E	600	67	3	1	1		
Sh4	Sandflies, dogs	Al Khanatar	22°42 N	58°32 E	400	87	5				
		Ibra	22°43 N	58°31 E						9	
Mascate	Patients, dogs	Muscat	23°32 N	58°24 E				1	1	4	
School	Patients, dogs	Kafaifa school	22°53 N	58°25 E				238	0		
Dh2	Sandflies	Djebel Al Qamar, rocks	16°55 N	53°44 E	20	24					
		Rocks	16°89 N	53°70 E	300	11					
Dh3	Sandflies, patient	Djebel Qara, farm	17°08 N	54°00 E	600	23	3	1	1		
		Djebel Qara, house, farm	17°07 N	54°01 E	500	25	2				
		Djebel Qara, rocks	17°06 N	54°03 E	240	20					
		Djebel Qara, rocks	17°06 N	54°03 E	240	30					
		Djebel Qara	17°14 N	54°02 E	800		2				
Dh5	Sandflies	Rocks	16°57 N	53°19 E	800	35					
		Jejouel, village	16°58 N	53°13 E	800	25					
Dh6	Sandflies, fox	Ouadi Herwouib, animal shelter	17°05 N	52°59 E	600	61	5				
		Ouadi Herwouib, rocks	17°04 N	52°59 E	600	34					1
Total						815	29	241	3	13	1

During the present field work, 21 stations were sampled in 18 localities using CDC traps (29 nights/traps) and/or the sticky trap (815 traps representing a total interception area of 65.2 m^2^). These stations were grouped by locality and/or biotope (Table 1).

Most of the sandflies were processed to be mounted *in toto* according to the following protocol. Soft tissues were lysed in a bath of KOH 10% (12 h), then washed four times in distilled water, cleared in Marc-André solution (12 h), and mounted individually between microscope slide and cover slide in Canada Balsam for species identification, after dehydration in successive alcoholic baths then clove oil.

A few specimens were processed individually to allow molecular biology processing [16, 19]. They were mounted in chloral gum directly after the Marc-André solution step.

Visual analysis of the specimens was performed by means of a BX61 microscope (Olympus, Japan). Measurements and counts were made using Stream Motion software (Olympus, Japan) and a video camera connected to the microscope.

The identifications were made thanks to the original descriptions of each of the species encountered, as well as available keys and papers [1, 10, 18, 39, 41–43].

### Vertebrate hosts sampling; *Leishmania* detection, isolation and identification

#### Human

##### Human leishmaniasis

Among the human leishmaniasis cases reported in Oman, only cutaneous forms were observed. All patients were hospitalised in Muscat and Ibra provinces (in Sharqiyah region) and Salalah province (in Dhofar region). Samples were biopsied by Arouette’s bistoury (punch biopsy of single use) under local anaesthesia of lidocaine (Xylocaine^®^). Samples were obtained by fine scissors or hooked pliers. After crushing the samples in Potter mortar in a sterile solution of NaCl (0.9%) plus penicillin G (200,000 u/mL), cultures were initiated on NNN medium (4 tubes per sample), with three drops of sterilised urine and a few drops of heart/brain solution, and incubated at 24 °C (23°–26 °C). Control was performed 5 days later and a subculture carried out every 8 days. Cultures were considered sterile after four subcultures.

Simultaneously, crushing on slides was performed using new cutaneous samples. Slides were fixed by methanol and stained by Giemsa; therefore, they were examined by direct microscopical examinations using 50× oil immersion objective.

Based on the leishmanin skin test method, the survey included a school population living close to Ibra. The *L. major* antigen, prepared by the *Istituto Superiore di Sanità* of Rome (Italy), was injected intradermally by Dermo-jet. Reactions were analysed 48 h later. The criterion for positivity was a papule of 5 mm diameter or more. Traditional leishmanin antigens react positively in most CL cases of the “Old World”, regardless of the *Leishmania* species.

#### Animal leishmaniases

We mainly focused on domestic canids (dogs) and wild canids (foxes). Animals were slaughtered under the control of the police services. The necropsies (one fox and 5 dogs) were carried out in veterinary centres (dogs) or in the field (foxes). Spleen, liver and bone marrow were targeted. Giemsa stain smears and cultures on NNN medium were performed according to the protocol detailed above for human leishmaniasis cases.

### *Leishmania* species identification

Isolated *Leishmania* strains were characterised by Multi Locus Enzyme Electrophoresis (MLLE) and confirmed by molecular identification. The identifications were performed at the *Leishmania* Identification Centre of the Unit of Vector-borne Diseases of the *Istituto Superiore di Sanità* of Rome (Italy).

### MLLE isoenzymatic identification

*Leishmania* strains isolated in the present study were typed by iso-enzyme analysis on starch-gel electrophoresis [60] of 15 enzymatic systems: malate dehydrogenase (MDH1), isocitrate dehydrogenase (ICD), phosphoglucomutase (PGM), fumarase hydratase (FH), 6-phosphogluconate dehydrogenase (PGD), glucose-6-phosphate dehydrogenase (G6PD), diaphorase (DIA1), glutamate dehydrogenase (GLUD), purine nucleoside phosphatase (NP1), purine nucleoside phosphorylase (NP2), glutamate-oxaloacetate transaminase (GOT1, GOT2), mannose phosphate isomerase (MPI), glucose phosphate isomerase (GPI), and malic enzyme (ME). The Omani *Leishmania* strains were typed using WHO reference strains of *L. infantum* zymodeme (Z) MON1 (MHOM/TN/80/IPT1), *L. donovani* ZMON2 (MHOM/IN/80/DD8)*, L. tropica* ZMON60 (MHOM/SU/74/K27) and *L. major* ZMON4 (MHOM/SU/73/5-ASKH). Furthermore, several strains of *Leishmania* species/zymodemes previously isolated in Oman’s neighbouring countries were also used as references. When a new zymodeme was identified, this was provisionally named a “variant” (var) of the most similar classified MON zymodeme, and the electrophoretic mobility (me) of the variant enzyme(s) was reported. The isoenzymes me were identified according to both Z Montpellier (MON) and Z Rome (ROM) nomenclatures.

### Molecular identification

The isolated *Leishmania* strains were typed by PCR-RFLP analysis targeting ITS-1 (internal transcribed spacer-1) [62] and Heat Shock Protein (HSP70) [70] according to the protocol detailed in the original techniques.

## Results

### Phlebotomine sandflies

During the present study, a total of 707 sandflies were captured by the technique of sticky traps. Of these, 331 were trapped in the Sharqiyah, with an overall density of 7.85 sandflies/m^2^ trap and 376 traps in Dhofar, at an average density of 16.32 sandflies/m^2^ trap (Table 2). Using the CDC miniature light traps, a total of 360 sandflies were captured: 115 sandflies in the Sharqiyah (with an average of 6.76 sandflies/night/trap) and 245 in the Dhofar (with an average of 20 sandflies/night/trap) (Table 3).

**Table 2:** Captures made using sticky traps in locations mentioned in Figures 1 and 2. ♂ = males, ♀ = females, ♂ + ♀ = males and females, s/m^2^ = density of sandflies (per m^2^ of sticky paper).

Location	Sharqiyah				Dh2				Dh3				Dh5				Dh6				Total Dhofar			
Species	♂	♀	♂ + ♀	s/m^2^	♂	♀	♂ + ♀	s/m^2^	♂	♀	♂ + ♀	s/m^2^	♂	♀	♂ + ♀	s/m^2^	♂	♀	♂ + ♀	s/m^2^	♂	♀	♂ + ♀	s/m^2^
*Phlebotomus papatasi*	35	14	49	1.16																				
*Phlebotomus bergeroti*	2	2	4	0.09													45	4	49	0.84	45	4	49	2.13
*Phlebotomus duboscqi*		2	2	0.05																				
*Phlebotomus alexandri*	3		3	0.07													31	4	35	0.60	31	4	35	1.52
*Phlebotomus saevus*					4		4	1.43		4	4	0.51	2		2	0.42	1	1	2	0.03	7	5	12	0.52
*Phlebotomus sergenti*	8		8	0.19													7	3	10	0.17	7	3	10	0.43
*Grassomyia dreyfussi*		2	2	0.05		1	1	0.36														1	1	0.04
*Sergentomyia fallax*	4	4	8	0.19	27	30	57	20.36									22	50	72	1.23	49	80	129	5.60
*Sergentomyia cincta*	29	9	38	0.90													5	2	7	0.12	5	2	7	0.30
*Sergentomyia christophersi*	67	39	106	2.51	1	8	9	3.21		3	3	0.38					8	6	14	0.24	9	17	26	1.13
*Sergentomyia clydei*	5	4	9	0.21																				
*Sergentomyia tiberiadis*	50	21	71	1.68	2	8	10	3.57	1	6	7	0.89	1		1	0.21	52	26	78	1.34	56	40	96	4.17
*Sergentomyia africana*																								
*Sergentomyia baghdadis*	13	12	25	0.59																				
*Sergentomyia (Sintonius) sp.*	1	2	3	0.07	1		1	0.36													1		1	0.04
Undetermined	3		3	0.07	1		1	0.36		1	1	0.13					4	4	8	0.14	5	5	10	0.43
Total	220	111	331	7.85	36	47	83	29.64	1	14	15	1.91	3	0	3	0.63	175	100	275	4.71	215	161	376	16.32

**Table 3:** Captures made using CDC miniature light traps in locations mentioned in Figures 1 and 2. ♂ = males, ♀ = females, ♂ + ♀ = males and females, s/n/t = number of sandflies per night per trap.

Location	Sharqiyah				Dh3				Dh6				Total Dhofar			
Species	♂	♀	♂ + ♀	s/n/t	♂	♀	♂ + ♀	s/n/t	♂	♀	♂ + ♀	s/n/t	♂	♀	♂ + ♀	s/n/t
*Phlebotomus papatasi*	3	13	16	0.94												
*Phlebotomus bergeroti*	1	4	5	0.29	1		1	0.14	57	27	84	16.8	58	27	85	7.08
*Phlebotomus duboscqi*																
*Phlebotomus alexandri*									15	16	31	6.2	15	16	31	2.58
*Phlebotomus saevus*					34	18	52	7.43	1	2	3	0.6	35	20	55	4.58
*Phlebotomus sergenti*																
*Grassomyia dreyfussi*																
*Sergentomyia fallax*					4	2	6	0.86	9	22	31	6.2	13	24	37	3.08
*Sergentomyia cincta*		2	2	0.12	1		1	0.14					1		1	0.08
*Sergentomyia christophersi*	4	5	9	0.53	4	2	6	0.86	5	6	11	2.2	9	8	17	1.42
*Sergentomyia clydei*	3	1	4	0.24	1		1	0.14					1		1	0.08
*Sergentomyia tiberiadis*		1	1	0.06	6	2	8	1.14	3	3	6	1.2	9	5	14	1.17
*Sergentomyia africana*										2	2	0.4		2	2	0.17
*Sergentomyia baghdadis*	25	53	78	4.59												
*Sergentomyia (Sintonius) sp.*																
Undetermined					1		1	0.14		1	1	0.2	1	1	2	0.17
Total	36	79	115	6.76	52	24	76	10.9	90	79	169	33.8	142	103	245	20

The species we caught belonged to the genera *Phlebotomus*, *Sergentomyia* and *Grassomyia*: *Phlebotomus* (*Phlebotomus*) *papatasi* (Scopoli, 1786), *Ph*. (*Phl*.) *bergeroti* Parrot, 1934, *Ph*. (*Phl*.) *duboscqi* Neveu-Lemaire, 1906, *Ph*. (*Paraphlebotomus*) *alexandri* Sinton, 1928, *Ph*. (*Par*.) *saevus* Parrot & Martin, 1939, *Ph*. (*Par*.) *sergenti* Parrot, 1917, *Sergentomyia* (*Sergentomyia*) *fallax* (Parrot, 1921), *Se*. (*Ser*.) *baghdadis* (Adler & Theodor, 1929), *Se*. (*Ser*.) *cincta* (Parrot & Martin, 1944), *Se*. (*Sin*.) *christophersi* (Sinton, 1927), *Se*. (*Sin*.) *clydei* (Sinton, 1928), *Se*. (*Sin*.) *tiberiadis* (Adler, Theodor & Lourie, 1930), *Se*. (*Parrotomyia*) *africana* (Newstead, 1912), and *Grassomyia dreyfussi* (Parrot, 1933). A few specimens were not identified (damaged) or identified to a subgeneric level (low mounting quality).

### Comments on sandfly species

The terminology used in this paper is that recently proposed [24].

#### *Phlebotomus (Phlebotomus) papatasi* (Scopoli, 1786)

The male is characterised by the presence of two spines at the end of the surstyle, by a group of more than ten big setae at the distal part of the gonocoxite, by the upper part of the paramere longer than the other ones and covered with setae along its full length. The ascoids are relatively short and never reach the next articulation.

The female is identified by its annealed spermathecae with sessile head wrapped in a cloud. Its pharyngeal armature presents teeth with, at the anterior part, many comb-like ones. Ascoids never reach the next articulation.

The distribution of this major vector of *L. major* [34] is very large, from Bangladesh to Morocco and from Crimea to Sudan. Limited to the North of the Sahara in West Africa, the species is most southern in East Africa. In Oman, the species is absent in the Dhofar, but dominates in the Sharqiyah. It was previously recorded in the Wahiba sands of the Sultanate [36].

#### *Phlebotomus (Phlebotomus) bergeroti* Parrot, 1934

The male is characterised by the presence of two spines at the end of the surstyle. The gonocoxite has a subapical tuft not exceeding ten setae. The upper part of the paramere is slightly longer than the other ones and covered with bristles in its distal half only.

The female is identified by its annealed spermathecae with sessile head, by a pharynx armed with teeth without spines or denticles on the posterior part and by the presence of anterior bilateral teeth. Ascoids reach or exceed the next articulation.

The distribution of *Ph. bergeroti* is wide: from Morocco to Iran. Its southern limit is the Sudan. In Oman, it is a scarce species in the Sharqiyah and the semi-arid zone of Dhofar, but it becomes abundant in the perarid part of the latter region. The strong anthropophily of *Ph. bergeroti*, and its abundance in certain perarid areas [2, 44] are arguments in favor of a significant vector role for *L. major* in the extreme deserts of Africa and the Arabian peninsula.

#### *Phlebotomus (Phlebotomus) duboscqi* Neveu-Lemaire, 1906

The male is characterised by the presence of four to seven spines at the end of the surstyle. The gonocoxite has about ten setae in its distal half, and usually two ones in its proximal part. The upper part of the paramere is slightly shorter than the other ones and is covered with setae throughout its length. The female is identified by its annealed spermathecae with sessile head. Its pharyngeal armature does not exhibit comb-like or lateral teeth. Ascoids sometimes reach the next articulation.

Its distribution is south of the Sahara to the Equator in Africa, and extends into the Arabian Peninsula. In Oman, the species is absent from Dhofar and seems rare in Sharqiyah, where we captured only two specimens.

This species constitutes in some foci a good alternative to *L. major* transmission by *Ph. papatasi* [15].

#### *Phlebotomus (Paraphlebotomus) sergenti* Parrot, 1917

The male has, like all *Paraphlebotomus*, a basal lobe on gonocoxite and its gonostyle carries four spines. However, it is easily identified by the curved shape of this basal lobe and by the brush of some setae that it carries, by its hooked parameral sheath, and by its globular gonostyle.

The female of *Ph. sergenti* is very difficult to separate from that of *Ph. saevus*. In *Ph. sergenti*, the well-developed pharyngeal armature contains strong elongated teeth, which are less numerous than in *Ph. saevus*. The geographical distribution of *Ph. sergenti* is very wide: from the Canary Islands to India and from Ukraine to Kenya. However, the diagnosis is delicate with an affine species *Ph. similis* whose distribution area that was initially thought to be limited to the North-east of the Mediterranean basin [17] is finally greater with a large area of sympatry in the Middle-East [47]. In Oman, we identified *Ph. sergenti* in small numbers (10 males) and still in wild sites (cavities and rocky chaos) in the Sharqiyah (Wadi Mouqal, at altitudes ranging from 550 to 600 m) and in the Dhofar (Wadi Herwouib, altitude 600 m). This species had already been captured in the north-east of the country, near the village of Awabi [61].

*Ph. sergenti* is the most important proven vector of *L. tropica* [4, 28].

#### *Phlebotomus (Paraphlebotomus) saevus* Parrot & Martin, 1939

The male of *Ph. saevus* has a straight, non-hooked parameral sheath, a large basal lobe of the gonocoxite, with a weakly dilated distal portion carrying many long and slightly curved setae.

**Figure 4:**
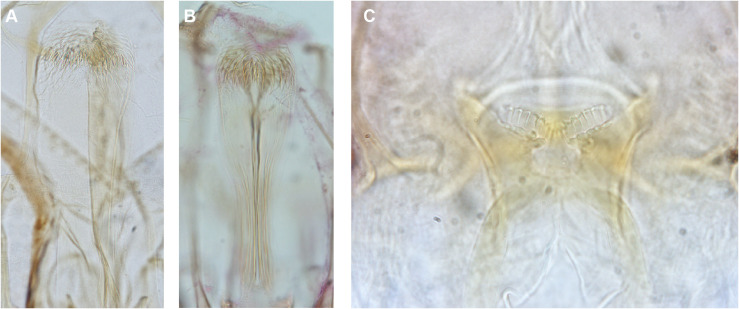
Pharynx of *Ph. saevus* (A), *Ph. sergenti* (B) and cibarium of *Se. baghdadis* (C).

The *Ph. saevus* female is difficult to distinguish from that of *Ph. sergenti*. Its pharyngeal armature is well developed and contains more teeth than those of *Ph. sergenti* (Fig. 4).

*Ph. saevus* has a distribution including East Africa and Arabia. In Oman, this is its first record. We caught *Ph. saevus* only in Dhofar (Djebel Quara), at the Dh3 capture site, an isolated farm where a female patient with leishmaniasis caused by *L. tropica* (LCO 4) lived.

*Ph. saevus* is a vector suspected of transmitting *L. tropica* in households where *Ph. sergenti* is absent, like in Kenya [45] or Yemen [14].

#### *Phlebotomus (Paraphlebotomus) alexandri* Sinton, 1928

The male and female are easily identifiable thanks to their short first flagellomere (= AIII).

Moreover, in the male, there is a short basal lobe of the gonocoxite, with a spherical head, provided with radiant, generally rectilinear setae. The apical spine of the style is inserted on a long process, far from the subapical spine.

The female exhibits a pharyngeal armature of rectangular overall appearance without anterior extension, consisting of strongly chitinised, spiniform scales forming a thick network.

*Ph. alexandri* occupies a vast geographical area: from Morocco to Mongolia down to Sudan. In Oman, *Ph. alexandri* is a fairly abundant *Phlebotomus*, especially in Dhofar. Here, *Ph. alexandri* is mainly found in the desert zone at *Boswellia* (Incense Tree), and more particularly in bottom of the Wadi (Herwouib).

With the exception of the isolation of *L. donovani* in China [27], the role of *Ph. alexandri* as a vector is still under discussion. Its low abundance in the prospected areas of the Sharqiyah cannot yet explain its potential role in the transmission of *L. donovani*.

#### *Grassomyia dreyfussi* (Parrot, 1933)

This species is distinguished from other Oman species by the absence, in both sexes, of ascoids on the first flagellomere, which is a characteristic of the genus *Grassomyia*. Moreover, both male and female harbour strong spines on each femur (pro- and meso- and metafemur).

The female is recognised by the very characteristic pattern of her spermathecae, in capsule of opium poppy.

The distribution of *Gr. dreyfussi* extends from Morocco to Iran and goes down to Kenya. It has recently been recorded in the Arabian Peninsula [20]. Its record in Oman is not surprising in this context. In the Sultanate, we have captured very rare specimens in the Sharqiyah and Dhofar.

Its role in the transmission of a *Leishmania* has never been mentioned.

#### *Sergentomyia* (*Sergentomyia*) *fallax* (Parrot, 1921)

The male genitalia has a long and narrow gonostyle with a non-deciduous silk implanted very distally.

The female has a large pharynx with a well-developed armature consisting with monomorphic teeth forming a heart-shaped pattern. The cibarium is armed with 15–23 pointed teeth, equal or sub-equal, arranged in an arch. The sclerotised area (= pigment patch) is oval.

The distribution of *Se. fallax* is wide. It extends from the Canary Islands and Morocco to Pakistan, covers the Arabian Peninsula and remains north of the Sahara. In Oman, *Se. fallax* is abundant in the Dhofar, while it is rather rare in the Sharqiyah.

The role of this species has never been mentioned in the transmission of a *Leishmania*, despite its vicariant *Se. dubia* being a possible vector of *L. infantum* in Senegal [68].

#### *Se*. (*Ser*.) *cincta* (Parrot & Martin, 1944)

This species belongs to the “Fallax group”. The male cannot be distinguished from those of *Se. antennata*. The female of *Se. cincta* exhibits fewer cibarial teeth (<20) than *Se. antennata* (>22).

*Se. cincta* is mainly distributed throughout eastern Africa, but has also been reported in West Africa [1, 67, 69]. It was recently found in Cameroon [69]. Taking into consideration the number of cibarial teeth as a valid specific character, we identified the female specimens of Oman as *Se. cincta* and associated males, pending revision of this group, using molecular tools to check whether *Se. cincta* is individualised from *Se. antennata*.

This is the first record of *Se. cincta* in the Arabian Peninsula. In Oman, we recorded it mostly in the Sharqiyah, and a few specimens in the Dhofar.

#### *Se*. (*Sintonius*) *christophersi* (Sinton, 1927)

As a member of the subgenus *Sintonius*, the male exhibits a pointed parameral sheath, whereas the female has annealed spermathecae, a common character in the genus *Phlebotomus* but an original character in the genus *Sergentomyia*, shared only by the members of the subgenus *Trouilletomyia* [55].

The identification of the male is based on a few teeth (3–7) of the cibarial armature and the existence of a row of a few vertical teeth. Similarly, the female exhibits a few cibarial teeth (2–5) and a few anterior vertical teeth (4–6) arranged along a line.

The distribution area of *Se. christophersi* is wide: from Morocco to India, including Cameroon [69] and the Arabian Peninsula [20, 42]. In Oman, we found *Se. christophersi* both in the Sharqiyah and in the Dhofar.

#### *Se*. (*Sin*.) *clydei* (Sinton, 1928)

As a member of the subgenus *Sintonius*, the male exhibits a pointed parameral sheath, whereas the female has annealed spermathecae.

The identification of the male is based on the presence of 16–35 small cibarial teeth. The female exhibits a row counting 10–15 cibarial teeth and a row of vertical teeth in variable number (from 4 to about 20) [19].

The distribution of *Se. clydei* is wide and was recently revised [19]: from Senegal to Afghanistan, through the Arabian Peninsula and the Seychelles.

In Oman, we recorded a limited number of specimens, more in in the Sharqiyah than in the Dhofar.

*Se. clydei* is a sandfly feeding on humans as well as on reptiles [1, 68] but no *Leishmania* vectorial role has been demonstrated for this species.

#### *Se*. (*Sin*.) *tiberiadis* (Adler, Theodor& Lourie, 1930)

As a member of the subgenus *Sintonius*, the male exhibits a pointed parameral sheath, whereas the female has annealed spermathecae.

The identification of the male is based on the presence of one row of 10–15 cibarial teeth, the median ones smaller than the lateral ones and 6–10 anterior vertical teeth. The female exhibits one row of 10–20 cibarial teeth, the median ones smaller than the lateral ones and two anterior rows of vertical teeth.

*Se. tiberiadis* is a species from the Middle East, including the Arabian Peninsula. It has never been involved in the transmission of *Leishmania*. It was previously recorded in the Wahiba sands [36].

#### *Se*. (*Parrotomyia*) *africana* (Newstead, 1912)

The female has a well-developed palisade-like cibarial armature of 55–80 teeth and smooth elongated capsule-like spermathecae typical of the subgenus *Parrotomyia*.

The male shows a cibarial armature of 20–35 teeth, palisade-like.

*Se. africana* is a member of a species complex called the Africana group, which requires revision by molecular tools as some identifications refer to the group rather than to the species *sensu stricto*. Its distribution area is wide. It includes Africa and the Middle-East, including the Arabian Peninsula.

This species has never been reported to be involved in the transmission of *Leishmania*.

#### *Se*. (*Par*.) *baghdadis* (Adler & Theodor, 1929)

The male can be identified thanks to its cibarium with angle-shaped notch and 14–16 teeth. The identification is easy thanks to the deep notch on the cibarium, also exhibiting about 30 teeth (Fig. 4).

Its distribution is limited to a zone ranging from Iraq to India). The record in Oman is the first in the Arabian Peninsula. We recorded it only in the Sharqiyah, not in the Dhofar. It has never been suspected of transmitting human *Leishmania*.

### Leishmaniases and *Leishmania* species identification

#### Skin tests

A total of 213 students (114 boys and 99 girls) from the school of Kafaïfa (Ibra province) underwent skin tests. Among them, 17 (8%) were positive. They exhibited an erythematous and pruritic papule of 1 or more cm in diameter. Girls were more susceptible to the disease (13.3%) than boys (10.1%). A low positivity rate seems to be due to a weak interaction between the young population and the *Leishmania* parasite in this focus (20 km from Ibra). It could be related to low density of sandfly vectors or their weak anthropophilia.

#### Human CL case reports, sampling and cultures

Four suspected human CL cases were observed, and 3 *Leishmania* strains were cultured and identified.

Case LCO 1:

Female, 17 years old, from the Muscat area (Sharqiyah region). Lesion on the right leg, beginning two years before. The CL diagnosis was confirmed by the presence of amastigotes in the lesion by microscopical examination. Treatment by intramuscular sodium stibogluconate (Pentostam^®^). Improvement but relapse. New hospitalisation for skin graft. Currently pretibial lesion: round-shaped ulcer of 4 cm diameter with a red/purplish elevated border and necrotic surface. Intra-lesional treatment by sodium stibogluconate (Pentostam^®^). In 1998, on September 28, biopsy under local anaesthesia. Direct examination: negative, *in vitro* culture on NNN medium: positive at the 2nd subculture. Strain MHOM/OM/98/IBM-103-ISS1783. MLLE identification: *L. donovani* ZMON-31 (= ZROM-24), *L. donovani* species identification was confirmed by molecular analyses.

**Figure 5:**
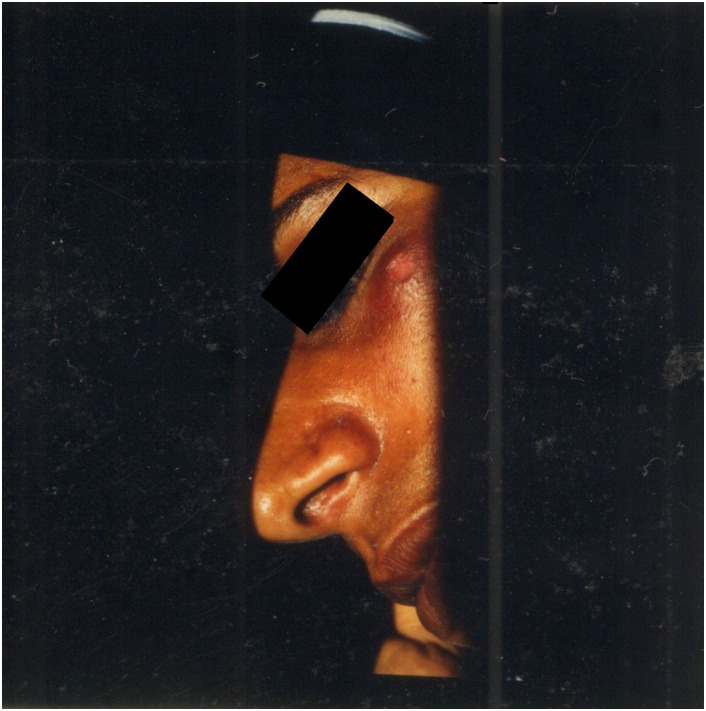
Ulcero-scabby lesion of case LCO 2.

Case LCO 2:

Female, 35 years old, living in Rowda (Ibra province, Sharqiyah region). Ulcero-scabby lesion on the left cheekbone, 3 cm in diameter with ulcerated centre and with an elevated and infiltrated border of 3–4 cm. In 1998, on September 16, consultation at Ibra hospital. CL diagnosis confirmed parasitologically by Giemsa stained smear. Local cryotherapy (pregnant woman). In 1998, on October 4, biopsy (Fig. 5). Direct microscopical examination of cutaneous smear and *in vitro* culture on NNN medium: positive. Strain MHOM/OM/98/IBM-104-ISS1790. MLLE identification: *L. donovani* ZMON-31 (= ZROM-24), *L. donovani* species identification was confirmed by molecular analyses.

Case LCO 3:

Boy, 12 years old, observed at Kafaïfa school, 20 km North of Ibra (Sharqiyah region). In the middle of the forehead, scabby lesion of 1 cm diameter, initiated 3 months before. Leishmanin skin test positive (1.5 cm). Cutaneous biopsy: direct microscopical examination and *in vitro* culture in NNN medium: negative.

Case LCO 4:

Girl, 4 years old, living in Ghadow on the southern slopes of Djebel Qara (Dhofar region), altitude: 500 m. The disease began 7 months before, by an erythematous lesion at the top of the nose. Treatment by intramuscular sodium stibogluconate (Pentostam^®^, 10 injections) was initiated on September 9. Examination at Salalah hospital on October 21: red and purplish infiltrated lesion (2 cm) at the top of the nose. Sample obtained by scraping with curette. Direct microscopical examination and *in vitro* culture in NNN medium: positive. Strain MHOM/OM/98/IBM-105-ISS1793. MLLE identification: *L. tropica* ZROM-102 (MDH100, ME100, ICD100, PGD95, G6PD85, GLUD100, DIA100, NP(1)450, NP(2)100, GOT(1)135, GOT(2)90, PGM100, FH100, MPI101, GPI76. The ZMON more similar was MON-39 with 4 variable isoenzymes (ME100, G6PD85, GLUD100, NP(2)100). The *L. tropica* species identification was confirmed by molecular analyses.

#### Animal leishmaniases

No *Leishmania* strain was isolated from canids.

## Discussion

The results presented in this work were collected in 1998 and have not been published until now. However, no data related to the Sultanate of Oman have been published since this field work [5]. Dating back more than 20 years, these data remain particularly interesting in this poorly explored region regarding leishmaniases and Phlebotomine sandflies.

There are some data related to sandflies in Oman in the literature. In the 1980s, a work focusing mainly on the sandflies of Saudi Arabia [40] reported a batch of 14 sandflies collected around Muscat including *Ph. alexandri*, *Se. fallax*, *Se. tiberiadis* as well as atypical specimens possibly belonging to new taxa close to *Se. christophersi* and *Se. schwetzi*.

Our sampling showed the presence of three species of *Paraphlebotomus*, whereas the subgenera *Larroussius*, *Synphlebotomus* and *Euphlebotomus*, confirmed or putative vectors of the *L. donovani* complex, are absent. The hypothesis of their role will be discussed later.

The detail of captures by province highlights the differences between the Sharqiyah and Dhofar (Tables 2 and 3). Thus, *Ph. papatasi* is observed mainly in the Sharqiyah, while *Ph. bergeroti* dominates in the Dhofar. Such distribution is also observed for *Ph. saevus* and *Ph. alexandri*, species well represented in Dhofar and rare or absent in the Sharqiyah.

The CDC miniature light traps positioned in the villages were of little or no yield in areas with a strong anti-malarial structure applying long-life insecticides regularly. Thus, in several villages of the Sharqiyah (Batem, Hedna, Rawda) where leishmaniases have been detected, the CDC remained systematically empty, in contrast to the sticky traps positioned in the wild sites, a few kilometres away from the CDC traps. The study carried out in the Dhofar confirmed this observation.

### Comments on *Leishmania* species identification

#### *Leishmania (Leishmania) tropica* (Wright, 1903)

The finding and isolation of *L. tropica* in Oman is not surprising. This parasite suspected of being responsible for CL [64], was recently isolated in Muscat from a Pakistani resident [65]. Moreover, several countries in the Middle East (Yemen, Saudi Arabia, Jordan, Syria, Iraq, Lebanon, Iran, Pakistan) and East Africa (Ethiopia, Kenya, Sudan) are known foci of *L. tropica* CL [8, 13, 53]. In neighbouring Yemen, there is an increasing trend of *L. tropica* CL [31–33]. Our results show the strain we isolated in the present study is related to Middle Eastern strains confirming the high polymorphism of *L. tropica* species and the description of a new variant zymodeme ROM102 (ZMON39 cluster).

The *L. tropica* isolation in a young girl who had never left the Dhofar region (case LCO 4, strain IBM-105) suggests the hypothetic vectorial role of *Ph. saevus*, the only *Paraphlebotomus* well represented on the site with 56 specimens caught (Tables 2 and 3), and the only member of the genus *Phlebotomus*, except one male of *Ph. bergeroti*.

#### *Leishmania (Leishmania) donovani* (Laveran et Mesnil, 1903)

The record of *L. donovani* ZMON-31 in Oman leads us to briefly discuss the taxonomic status and the geographical distribution of this zymodeme and closely related zymodemes.

Starting in the 1980s, enzymatic taxonomy studies led to consider the linnean taxa *L. donovani* and *L. infantum* as two distinct phenetic groups. Cladistic analysis confirmed these results by showing their monophyly [37, 48, 60]. In fact, these phylogenetic groups (or complex) possessed a series of synapomorphic states, such as G6PD 100, G6PD 102 G6PD 105, GPI 86, GPI 100, GOT1 100, GOT2 100, GOT 113, and GOT2 113. Some of these states were common to both branches, others only present in one of them. This was the case of PGM 100, present in the *donovani-infantum* set (complex synapomorphies), GOT1 100 and GOT2 100 present in the single subset *L. infantum*, and GOT1 113 and GOT2 113 present in the single subset *L. donovani* (specific synapomorphies). The taxonomic status of the *donovani-infantum* group changed at the end of the 1980s. Enzymatic analysis of human *Leishmania* strains isolated in Sudan [49, 60] and the vector *Ph. orientalis* [11] showed an original zymodeme (MON-82*, L. archibaldi*) characterised by a heterozygous structure for GOT1 (100/113) and GOT2 (100/113), and highlighted the complexity of the systematics of *L. donovani* s. l. [54]. Moreover, hybrids could develop differently in sandfly vectors [66].

*L. donovani* ZMON-31, isolated from CL in Oman, was already known from Saudi Arabia [51] and Yemen [56] as responsible for VL. Moreover, this zymodeme is phylogenetically closely related to *L. donovani* ZMON-83 (Ethiopia), ZMON-3 (Iraq), ZMON-37 (Kenya), and ZMON-2 (India), all responsible for VL. Finally, a new zymodeme, *L. donovani* MON-191, close to ZMON-31, was isolated from a French tourist traveling to Yemen contracting a CL [52]. Multilocus microsatellite typing (MLMT) revealed genetically isolated populations in the main endemic VL regions [35]. *Leishmania donovani* ZMON31 was identified within the predominantly anthroponotic *L. donovani* cluster of Sudan/Ethiopia.

Regarding the clinical aspect, even though *L. donovani* s.st. is poorly studied for tissue tropism, the complex *L. donovani–L. infantum* appears to cause both CL and VL.

Therefore, we consider *L. donovani* s.st. as the most probable agents of VL in Oman. However, *L. infantum s. st.* would be in second place, if its presence should be confirmed, as it is in Yemen (Taëz), a country where *L. donovani*, *L. infantum* and *L. tropica* are sympatric [57]. In Oman, the scarcity of dogs, domestic or feral, in relation to the significant number of reported human cases seems more in agreement with the circulation of *L. donovani* than that of *L. infantum*. On the basis of the sandflies collected, the proven or candidate vectors of *L. donovani* were not recorded: *Ph.* (*Eup.*) *argentipes* [34], *Ph*. (*Lar*.) *orientalis*, *Ph*. (*Syn*.) *celiae*, *Ph*. (*Syn*.) *martini*, or *Ph*. (*Ana*.) *rodhaini* [3]. Consequently, the vector candidates for *L. donovani* transmission in the Sharqiyah region still remain unknown. For us, the only candidate could be *Ph. alexandri*, a species suspected in China [27] or in Cyprus [9, 38], but the low densities of this species observed in Oman do not argue in favour of any role.

The low positivity rate of the skin test (8%) reported in children of Ibra province suggests a weak interaction between the young population and the *Leishmania* parasite, confirming a low density of sandfly vectors or their weak anthropophilia.

Consequently, in order to better understand the epidemiology of the leishmaniases in Oman, we encourage the isolation and typing of *Leishmania* strains in the future, because each *Leishmania* complex often corresponds to a specific vector and to a particular parasitic cycle (anthroponosis/zoonosis). This was the problem previously raised when *L. infantum* was suspected to be the causative agent of VL in Oman [30], whereas *L. donovani* s.l. was later incriminated as the agent of VL [63].

#### Conflict of interest

None of the authors of this manuscript have a commercial or other interest that might represent a conflict.
